# In Situ Growth of Halophilic Bacteria in Saline Fracture Fluids from 2.4 km below Surface in the Deep Canadian Shield

**DOI:** 10.3390/life10120307

**Published:** 2020-11-24

**Authors:** Regina L. Wilpiszeski, Barbara Sherwood Lollar, Oliver Warr, Christopher H. House

**Affiliations:** 1 Department of Geosciences and Earth and Environmental Systems Institute, The Pennsylvania State University, University Park, PA 16802, USA; ginawilp@gmail.com; 2 Stable Isotope Laboratory, University of Toronto, Toronto, ON M5S 3B1, Canada; barbara.sherwoodlollar@utoronto.ca (B.S.L.); oliver.warr@utoronto.ca (O.W.)

**Keywords:** subsurface biosphere, deep life observatory, microbial diversity, groundwater

## Abstract

Energy derived from water-rock interactions such as serpentinization and radiolysis, among others, can sustain microbial ecosystems deep within the continental crust, expanding the habitable biosphere kilometers below the earth’s surface. Here, we describe a viable microbial community including sulfate-reducing microorganisms from one such subsurface lithoautotrophic ecosystem hosted in fracture waters in the Canadian Shield, 2.4 km below the surface in the Kidd Creek Observatory in Timmins, Ontario. The ancient groundwater housed in fractures in this system was previously shown to be rich in abiotically produced hydrogen, sulfate, methane, and short-chain hydrocarbons. We have further investigated this system by collecting filtered water samples and deploying sterile in situ biosampler units into boreholes to provide an attachment surface for the actively growing fraction of the microbial community. Scanning electron microscopy, energy-dispersive X-ray spectroscopy, and DNA sequencing analyses were undertaken to classify the recovered microorganisms. Moderately halophilic taxa (e.g., *Marinobacter, Idiomarina, Chromohalobacter, Thiobacillus, Hyphomonas, Seohaeicola*) were recovered from all sampled boreholes, and those boreholes that had previously been sealed to equilibrate with the fracture water contained taxa consistent with sulfate reduction (e.g., *Desulfotomaculum)* and hydrogen-driven homoacetogenesis (e.g., *Fuchsiella*). In contrast to this “corked” borehole that has been isolated from the mine environment for approximately 7 years at the time of sampling, we sampled additional open boreholes. The waters flowing freely from these open boreholes differ from those of the long-sealed borehole. This work complements ongoing efforts to describe the microbial diversity in fracture waters at Kidd Creek in order to better understand the processes shaping life in the deep terrestrial subsurface. In particular, this work demonstrates that anaerobic bacteria and known halophilic taxa are present and viable in the fracture waters presently outflowing from existing boreholes. Major cations and anions found in the fracture waters at the 2.4 km level of the mine are also reported.

## 1. Introduction

Taking advantage of the groundwaters of the deep hydrogeosphere, the microbial biosphere extends into the fractures and pore spaces of the continental crust, one of the lowest energy environments and most isolated habitats on Earth. Deep fracture waters in the continental subsurface support independent, self-sustaining ecosystems driven by abiogenic hydrogen and lithoautotrophy [1,2,3,4]. A growing number of studies suggest that such ecosystems are widespread in the terrestrial subsurface, including evidence of chemoautotrophs in the fracture waters of deep groundwaters below Sweden, Finland, South Africa, Japan, and the United States (e.g., [5,6,7,8,9,10,11,12]). This environment serves as an analogue for planets with little tectonics and low seismic activity. The past surface habitability of Mars [13] has diminished over time [14], but liquid water in the subsurface has presumably persisted through to the present [15]. Mars has on-going low-level seismic activity [16] indicative of subsurface fracturing that likely augments fracture networks developed over the history of Martian geophysical and impact processes. Overall, the Kidd Creek fracture waters have many attributes that should be common among subsurfaces of terrestrial planets with slowly progressing fracture networks with little or no influx of surface fluids.

The presence of lithotrophs in the deep subsurface on Earth, along with isotope and chemical data, suggests that a deep biosphere can be sustained independently from surface photosynthesis [1,17,18]. These proposed ecosystems derive energy from hydrogen, sulfate, carbon dioxide, methane, and simple organic molecules that outgas from cooling magma or water–rock reactions, rather than from the sun [19,20]. They extend to at least 3–4 km below the surface [4,7,8,9,10,11,12,21] and have been estimated to contain 2 to 6 × 10^29^ cells [22].

**Figure 1:**
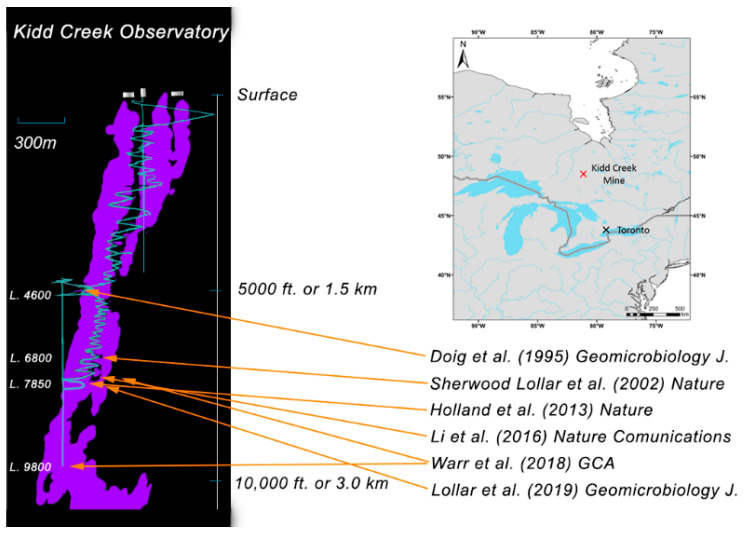
Study location: Kidd Creek Observatory in the Superior Province of the Canadian Shield north of Timmins, Ontario, Canada. Thin blue vertical lines represent the shaft and cage system for accessing the subsurface, while spiraling blue lines represent the ramp road descending from the surface to the Kidd Creek Observatory located at 2.4 km (L. 7850) below surface.

One such ecosystem has been described in fracture waters of the Kidd Creek Observatory located in Timmins, Ontario, in 2.7 billion year old rocks of the Canadian Shield [23] (Figure 1). Between 4–7 million years of episodic hydrothermal activity resulted in formation of a volcanogenic massive sulfide (VMS) deposit analogous to modern day black smoker systems on the ocean spreading centers [24]. Post-depositional deformation of the crust tilted the ore body (shown in purple) from the original sublateral (horizontal) position to the near vertical position today. Geochemical and geomicrobiological investigations at this location have been underway since the mid–1990s [25], with continuous monitoring of the boreholes at the 2.4 km observatory for more than 10 years. Characterization has been done of dissolved gases [26], noble gas residence times [23,27], and both indirect [17] and direct evidence [4] for indigenous microorganisms in the fracture fluid waters (Figure 1). Viable microbial biofilms were previously collected from Kidd Creek via in situ incubations in shallower boreholes (1402 m depth) for chemical and morphological descriptions, showing abundant bacterial cells with varying morphotypes in a matrix with significant quantities of inorganic material and extracellular polysaccharides [20,25]. More recently, cultivations and chemical analyses were used to recover low-biomass communities and evidence for viable sulfate-reducing microorganisms (dominantly autotrophic and alkane-oxidizing sulfate-reducing bacteria) in fracture fluids at Kidd Creek [4,21]. The 2019 study provides details of the geological setting and the hydrogeological, geochemical, and culture-based microbiological (most probable numbers analysis) results from long-term international studies that have been underway for over a decade to investigate this isolated component of the deep hydrogeosphere and terrestrial biosphere.

The current study focused on exploratory boreholes (300–600 m long) intersecting ancient saline fracture waters that discharge from the boreholes under artesian pressure. Noble gas measurements indicate that waters flowing out of the boreholes have mean residence times on the order of hundreds of millions of years to 1 billion years [8,9,10,11,12,23,27]. Isotopic evidence is consistent with the fracture fluids containing abiogenically derived hydrogen, methane, and sulfate in quantities theoretically sufficient to support microbial growth [15,17,26,28,29]. These geochemical processes present long-lasting sources of electron donors and electron acceptors energetically sufficient to support an ecosystem in the absence of photosynthetically derived inputs [17,18].

In this study, we investigated the microbial community found in fracture water from three boreholes at the 2.4 km depth at the Kidd Creek Observatory on the basis of DNA sequence analyses and electron microscopy. DNA extracted from filtered water samples provided information about the total community while in situ biosampler units were deployed to collect the actively growing fraction for identification and imaging. These data complement recent reports on the abundance and activity of the microbial community in this environment, elucidating the diversity of microorganisms on the basis of 16S ribosomal ribonucleic acid (rRNA) sequencing of the total and active fractions of the local microbial community.

## 2. Materials and Methods 

We collected samples from 3 boreholes at the 2.4 km level both by filtration of flowing fracture water to concentrate cells and/or by in situ incubation of a sterile biosampler unit to provide attachment surfaces for actively growing cells to adhere to. All 3 boreholes in this study (as well as those described in [4,17,23,27]) were from the same rock face in the Kidd Creek Observatory at 2.4 km (indicated in Figure 1). All were located within approximately 2–3 m of each other, and each extended into the rock for several hundred meters from that point. Detailed host rock lithologies are provided in Li et al. (2016). The fracture water (FW) from 12299 has been accessed regularly for scientific study since it was drilled in 2007 [4,27]. FW12299 has been isolated from the mine environment for approximately 7 years by a sterile high-quality stainless steel manifold that is resistant to alteration by microbial activity, analogous to the “CORKs” (circulation obviation retrofit kits) that often have been used in studies of deep-sea boreholes [1,30,31,32]. Before installation, the manifold was combusted at 400 °C for 8 h and autoclaved. Before sampling from the main valve, we opened 4 side valves to let the fracture water flow under natural high pressures for several minutes. This process flushed out any water that might have been oxygenated during the initial contact with mine air after opening the valve, and also flushed air out of the sterile manifold. Outflowing fracture fluids in FW12299 have an average temperature of 26 °C and are slightly acidic (pH ≈ 5.75) with anoxia, high conductivity, elevated H_2_ (>3 vol % in gas phase), and very high (>70 vol % in gas phase) methane concentrations attributed to abiotic methane production (Table 1) [17,26]. The recent most probable number (MPN) paper on this system that demonstrated this on the basis of both ^18^O and ^2^H values, along with the large purge volumes that have flowed from these boreholes since completion, both ensure that a return to baseline has been achieved and no contamination of fluids remains in the holes [4]. High water flow rates of 770 mL/min have declined somewhat over time, but for the past several years they have ranged between 100 and 150 mL/min (Table 1).

Fracture water FW12287A has also been accessed for scientific study on previous expeditions [17,27]. It too has water with a very long residence time and is similarly anoxic, enriched in methane (>70 vol %) and hydrogen gas (>10 vol %), and is highly saline (Table S1, in the supplementary). For this study, FW12287A was sampled for DNA by filtration of the flowing water naturally discharging from the borehole at rates between 79 and 5 mL/min (Table 1). Sampled water contained fine-grained dark sediment that rapidly clogged the filters used.

Fracture water FW12322 discharges from an open borehole with no packer and is open to the mine environment. While discharging fluids ensure the borehole is continuously flushed, flow rates are significantly less than for the other 2 boreholes and hence the fluids at the outlet may be impacted by contact with the aerobic mining environment [4]. The low flow rate for FW12322 in particular means it represents, among the boreholes studied, the one most likely to be impacted by contact with the mining environment. FW12322 had not been studied to the same extent as the other boreholes but it intersects the same rock layer, and thus the physical and chemical environment was assumed to be similarly saline, slightly acidic, and reduced with an average temperature of approximately 25 °C. 

### 2.1. Filtration of Mine Water

Cells were collected from 60 mL water samples onto 0.22 mm polyethersulfone syringe filters (MilliporeSigma, St. Louis, MO, USA) in the field. Filters were stored at 4 °C for transport and were frozen at −20 °C prior to DNA extraction. Additional unfiltered aliquots of water from FW12299 were collected in autoclaved glass bottles for use in method development and for controls. Mine water aliquots were maintained at 4 °C and 60 mL and were collected onto a 0.22 mm filter immediately prior to use as a process control for DNA extraction.

### 2.2. In Situ Incubation and Fixation

In situ incubations were collected on a nylon biosampler unit modified from the peeper design of House et al. [33]. The biosampler unit consisted of a 1-inch by 24-inch nylon base holding 12 1-inch diameter round slides (Figure S1, in the supplementary). Glass, tin oxide-coated glass, and silicon slides were exposed to the environment through a 0.9-inch diameter window that was protected by a nylon mesh screen to exclude large (>150 mm) particles and, for a subset of the slides, a 0.2 mm size-exclusion filter to exclude smaller particles. 

All nylon pieces were washed and autoclaved prior to assembly. Silicon wafers were visually inspected for contamination and deployed as is from the manufacturer. Glass and tin oxide-coated glass slides were washed with soap and water, rinsed with ultra-pure double-distilled water, acid washed by soaking in 3.6% HCl for 1 h, rinsed again with ultra-pure double-distilled water, and dried. The biosampler unit was assembled using flame sterilized forceps and tools within a positive pressure laminar flow hood. Autoclaved nylon rope was affixed to the unit for retrieval from the borehole. The assembled biosampler units were wrapped in autoclaved aluminum foil for transport to the mine.

Care was taken to avoid introducing contaminants into the boreholes while deploying the biosampler units. Pre-sterilized biosamplers were inserted approximately 1m into the interior of the borehole and were left in place for 200–230 days (Figure S2, in the supplementary). Prior to deployment, 12,299 was sealed with a pre-existing packer and tube system used to aid in chemical studies of the borehole, which was re-sealed after the sampler was inserted. 12,322 was previously open to the mine environment and was sealed with a sterilized rubber stopper with a small pressure-relief valve after deployment of the biosampler unit. 

Upon retrieving the biosamplers from the boreholes at Kidd Creek, we wrapped them in sterile aluminum foil, placed them into plastic bags, and shipped them back to Penn State on ice. Slides were removed from the biosampler using flame-sterilized forceps and fixed in a 2% paraformaldehyde solution in 1× phosphate-buffered saline (PBS) for 4 h. Fixed slides were rinsed and dehydrated in 1× PBS, 50/50 PBS/ethanol, and 100% ethanol. Dehydrated slides were stored individually at −20 °C. To avoid precipitates (Figure S3, we recommend using non-phosphate saline solutions for additional molecular biological research at Kidd Creek.

Cell-free slides for use as controls for downstream processing were prepared in the lab using water collected from FW12299 that was sterilized by filtration through a 0.22 mm syringe filter to remove cells and particulates. A total of 200 mL filtered water was added to the surface of each glass slide and air dried inside a sterilized laminar flow hood. The slides were fixed in a 2% paraformaldehyde (PFA) solution as described above and were processed through the analysis pipeline described below.

### 2.3. SEM and EDS

Four silicon wafers incubated in the fracture waters containing PFA-fixed, dehydrated in situ growth samples were analyzed by scanning electron microscopy (SEM) and energy-dispersive X-ray spectroscopy chemical analysis (EDS) at the Penn State Materials Characterization Lab (University Park, PA, USA) using a Nova NanoSEM 630. Samples were coated in 3 nm iridium to enhance conductivity prior to imaging. EDS spectra were collected from 0–5 keV, and images were compiled using the software package AZtec (Oxford Instruments, Concord, MA, USA), excluding the iridium coating signal from the resulting spectra.

### 2.4. DNA Extraction

DNA was extracted from silicon wafers using the Epicenter Masterpure kit (Lucigen, Middleton, WI, USA) following the manufacturer’s instructions with the following modifications: 300 mL Tissue and Cell Lysis Solution was used to sample cells from the surface of the slides into the solution for extraction. Then, 1 mL Proteinase K was added, tubes were vortexed for 30 s, and samples were incubated at 65 °C for 30 min, vortexing briefly every 10 min, before proceeding with the recommended protocol. A clean silicon wafer (extraction control) was fixed in PFA and subjected to the same extraction protocol. 

DNA was extracted from filters using the MoBio Power Biofilm DNA Isolation Kit (MoBio, San Diego, CA, USA) with the following procedure modified from the manufacturer’s instructions. Briefly, 3× filters from each borehole were processed in parallel with one another. Ethanol- and flame-sterilized pliers and forceps were used to remove the syringe filters from their casing and into the kit bead tubes. Then, 350 mL warm buffer BF1 and 100 mL BF2 were added and samples were briefly vortexed. Tubes were incubated 10 min at 65 °C, then vortexed horizontally 1 min at maximum speed. Samples were centrifuged 1 min at 13,000× *g* and the supernatant was transferred to clean tubes. After this, 200 mL BF3 was added, briefly vortexed, and then incubated on ice for 5 min. Samples were centrifuged for 1 min at 13,000× *g* and the supernatant was transferred to another clean tube, taking care to avoid the pellet. Then, 900 mL warm buffer BF4 was added and all 3 extracts were run through a single DNA-binding column per borehole to concentrate the DNA. The column was rinsed with 650 mL BF5, then 650 mL BF6, was centrifuged 2 min at 13,000× *g*, and eluted in 100 mL buffer BF7. An extraction control of a filter from nuclease-free water was processed alongside the sample extractions. All extracted DNA was stored at −20 °C prior to further use.

### 2.5. DNA Amplification and Sequencing

The V4 hypervariable region of the 16S rRNA gene was PCR-amplified from purified DNA using KAPA2G Robust HotStart ReadyMix (Kapa Biosystems, Wilmington, MA, USA). Negative PCR controls were processed in parallel with each round of amplification, and each DNA extraction was amplified and sequenced in duplicate. The universal bacterial and archaeal primer pair 515F (5′-GTGCCAGCMGCCGCGGTAA-3′)—806R (5′-GGACTACHVGGGTWTCTAAT-3′) was used, with Illumina Nextera adapters added to aid in downstream processing [34]. Each PCR tube contained 12.5 μL KAPA2G Robust HotStart ReadyMix, 8.5 μL nuclease-free H_2_O, 1.25 μL forward primer (10 mM), 1.25 μL reverse primer (10 mM), 0.5 μL 20 mg/mL bovine serum albumin (BSA), and 1 μL template DNA. The mixture was cycled as follows: 95 °C for 3 min; 35 cycles of 95 °C for 15 s, 60 °C for 15 s, 72 °C for 15 s; then 72 °C elongation step for 1 min. Amplicon size was confirmed by visualization on a 1% agarose gel stained with ethidium bromide. Multiplexed libraries were prepared and sequenced at the Penn State Genomics Core Facility (University Park, PA, USA) using the Nextera XT v3 300 × 300 nt kit for the Illumina MiSeq (Illumina, San Diego, CA, USA).

In terms of the extracted filter samples, the total biomass and resulting DNA concentration were low, and thus we added an additional whole genome amplification step in order to pre-amplify DNA prior to PCR amplification to avoid introducing extra bias from excessive PCR cycles. Whole-genome amplification was performed using the Repli-G Mini Kit (Qiagen, Germantown, MD, USA) following the manufacturers protocol and samples were stored at 4 °C until PCR amplification (<48 h) as described above.

The full length 16S rRNA gene was also sequenced for a subset of in situ growth samples to better understand the species-level diversity of recovered microbes. The full-length 16S rRNA gene (27F–1492R) [35] was PCR-amplified for sequencing as above but with changes to the cycling conditions: 95 °C for 3 min; 35 cycles of 95 °C for 15 s, 60 °C for 15 s, 72 °C for 55 s; then 72 °C elongation step for 2 min. Sample libraries were prepared and sequenced at the Penn State Genomics Core Facility (University Park, PA, USA) using PacBio Sequel SMRT-cell technology.

All unprocessed sequence data has been uploaded to the NCBI Sequence Read Archive under Bioproject PRJNA667171.

### 2.6. Genetic Analyses

Operational taxonomic units (OTUs) were generated from the V4 Illumina dataset using the program mothur v.1.36.1 following the published MiSeq SOP [36,37]. A total of 20,000 merged paired-end reads per sample were subsampled and screened for length and quality. Unique sequences were aligned to the MOTHUR-compatible SILVA SEED database (release 128). Aligned sequences were screened and filtered against the alignment. After a pre-clustering step, chimeras were identified and removed using the uchime algorithm implemented in mothur [38]. Remaining unique sequences were classified according to the Silva v128 database. Distances were determined using default options and sequences were clustered at the 3% similarity level using the opticlust algorithm. OTUs were classified by taxonomy and results were imported into R for further analysis. Species-level classifications were assigned by exporting representative consensus sequences for each OTU and comparing them to the RefSeq 16S Microbial Database [39] using a local installation of BLAST+ [40]. The top hit for each OTU was saved and the BLAST results were processed using MEGAN 6 [41]. 

Full length 16S rRNA data generated by Sequel sequencing were received in the form of barcode parsed consensus sequence bamfiles. The data were converted to fasta format using BEDtools and Seqtk [42]. Fasta-formatted sequences were treated as contigs and processed using mothur as described above.

## 3. Results and Discussion

### 3.1. SEM and EDS Results of In Situ Incubations

Biosamplers were retrieved after 200–230 days incubation within the boreholes. The returned biosamplers were coated in a thin layer of fine sediment (Figure S4, in the supplementary). After fixation with paraformaldehyde, the fixed slides were observed under 1000× magnification and found to be coated in a patchy precipitate layer (Figure S3, in the supplementary). In follow-on microscopy (SEM and EDS) of the slides, the precipitates could be avoided due to their patchy nature.

**Figure 2:**
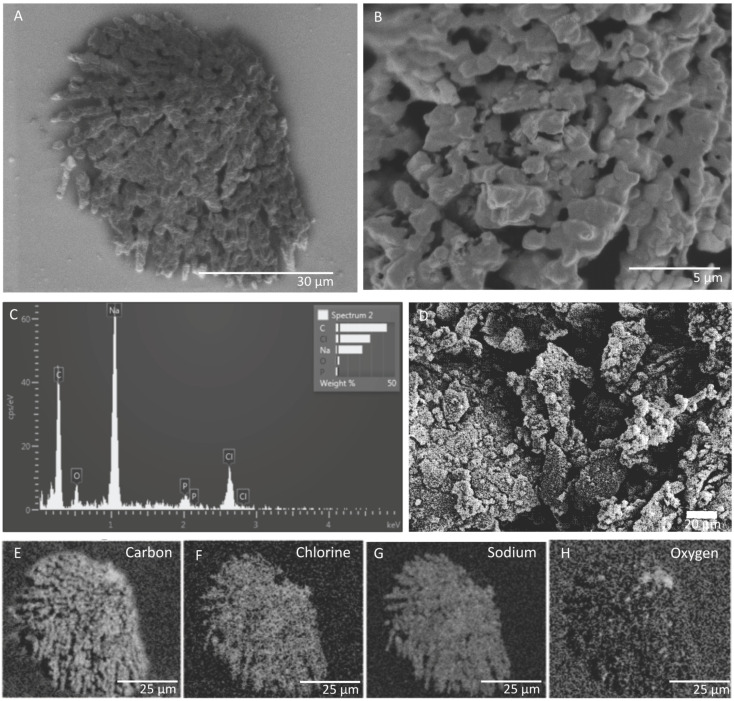
(**A**) SEM image and (**B**) detailed view of a biofilm feature in FW12299, which has been sealed from the mine environment via a packer or “CORK” (circulation obviation retrofit kit). (**C**) Energy-dispersive X-ray spectroscopy chemical analysis (EDS) spectrum of the biofilm. (**D**) Biofilm from hydrogen- and methane-rich waters from Copper Cliff South mine for comparison [25]; shown with permission from Taylor & Francis Ltd., www.tandfonline.com. (**E**–**H**) EDS imaging of elements identified within the biofilm as collected by secondary electron detector.

**Figure 3:**
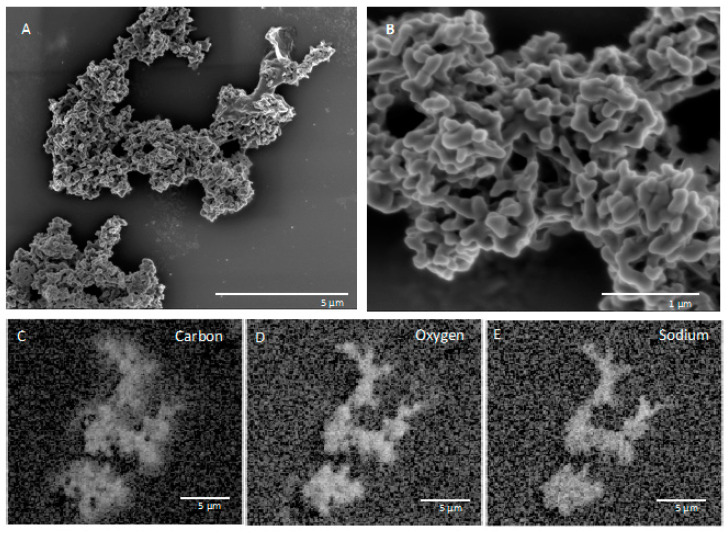
(**A**) SEM image and (**B**) detailed view of a second biofilm morphology from FW12299 with (**C**–**E**) EDS elemental imaging biofilm as collected by secondary electron detector.

SEM and EDS images of in situ growth surfaces on silicon wafers revealed carbon-rich structures consistent with biological growth and biomineralization. Multiple morphologies were observed. One structure on a slide from FW12299 was similar in texture to a biofilm previously observed at a shallower depth (1.2 km) in the Copper Cliff South mine in Sudbury, Ontario, located 290 km south of Kidd Creek (Figure 2) [25]. The previous study did not identify the elements associated with the biofilms or the genetic identity of growing cells, but in our samples EDS results showed that the cells grew in close association with sodium chloride. It is possible that the specific morphology is related to biomineralization of carbonate, which has been observed in studies of *Halomonas* species and *Chromohalobacter marismortui* [43,44], although the lack of calcium in association with the carbon suggests otherwise. Additional structures seen on the in situ surfaces incubated in FW12299 commonly appeared with similar structures to those in Figure 3. These structures were rich in carbon, oxygen, phosphorus, and sodium. 

Slides incubated in FW12322 had carbon-rich structures with morphologies different than those found in FW12299. Biofilms were less structured than those from FW12299, with carbon-rich regions co-occurring with calcium chloride crystals and displaying a fine scale structure similar to that observed in growth on slides from FW12299 (Figures S5 and S6).

### 3.2. Fracture Water vs. Service Water

Mine environments contain waters from the surface pumped into the mine for drilling and other mine operations (e.g., service water, SW). Previous studies have demonstrated that the δ^18^O and δD values for the boreholes investigated here are distinctly different from service water and hence can rule out a major contamination from this source in these fluids in the present day [4]. That said, as service water was the drilling fluid used at the time of drilling for all boreholes, the potential impact of surface-derived microorganisms in service water cannot be ignored. The 2019 microbiological study at Kidd Creek demonstrated that the metabolic response of cultivated organisms in a nearby borehole at the same depth (2.4 km) differed from the responses in the SW samples taken for comparison. Specifically, the service water had a substantial contribution from heterotrophic sulfate reducers while the fracture water samples were dominated by alkane-oxidizing sulfate reducers and autotrophic sulfate reducers, and the fracture water also had significantly lower cell counts compared to SW [4]. The dominance of autotrophic over heterotrophic sulfate-reducers in the fracture water is consistent with findings from other subsurface ecosystems at depths of 2–3 km investigated to date where autotrophic metabolic metabolisms dominate over heterotrophic metabolisms [4]. While this present study did not collect SW samples as environmental controls during this sampling effort, a separate group has identified the predominant taxa observed in service water from this area. Comparison of our results with theirs shows *Burkholderiaceae*, *Chromohalobacter*, *Cupriavidus*, and *Thiobacillus* as common taxa between the service water taxa (Sian Ford, Greg Slater, Katja Engel, and Josh Neufeld—personal communication) and the results reported here.

### 3.3. Results of 16S rRNA Sequencing

To identify specific taxa present within the boreholes, we extracted and sequenced the 16S rRNA gene from duplicate silicon wafers that were incubated in the boreholes. 16S ribosomal DNA was also sequenced from filtered water samples as well as negative controls for the DNA extraction and amplification processes to control for any low-level contamination that might be introduced by process or reagents while working with these very low biomass samples [45]. 

DNA extracted from the in situ growth surfaces for both boreholes FW12299 and FW12322 was readily amplified with no extra processing needed, but the filter samples contained very low biomass that yielded low DNA concentrations for FW12299 and FW12287A. DNA extractions from multiple filters were combined to generate enough product to sequence the filter samples, and whole-genome DNA was pre-amplified by Repli-G rolling circle amplification (Qiagen) prior to PCR amplification of the V4 hypervariable region of the 16S rRNA gene. 

**Figure 4:**
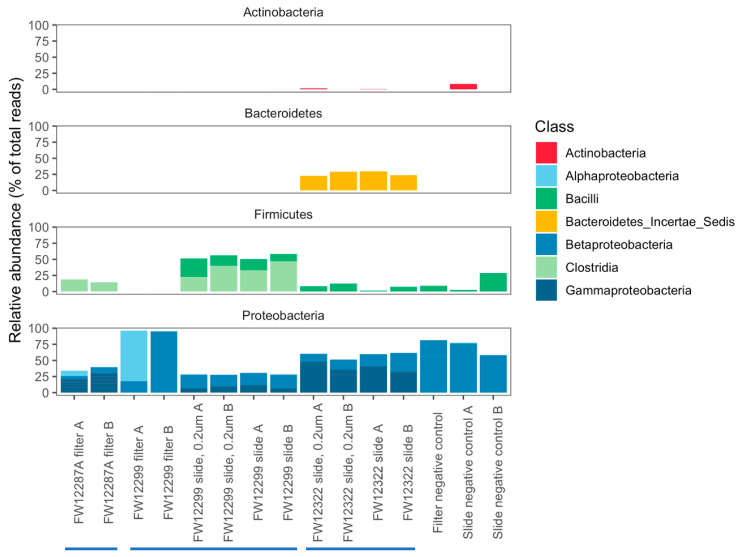
16S classification by phylum and class for abundant operational taxonomic units (OTUs) (those representing >0.5% total sequences) on the basis of the V4 hypervariable region of the 16S gene. Bacteroidetes were enriched in the borehole that was recently exposed to the mine environment (FW12322). Clostridia were enriched in boreholes sealed from the mine environment (FW12887A and FW12299).

**Figure 5:**
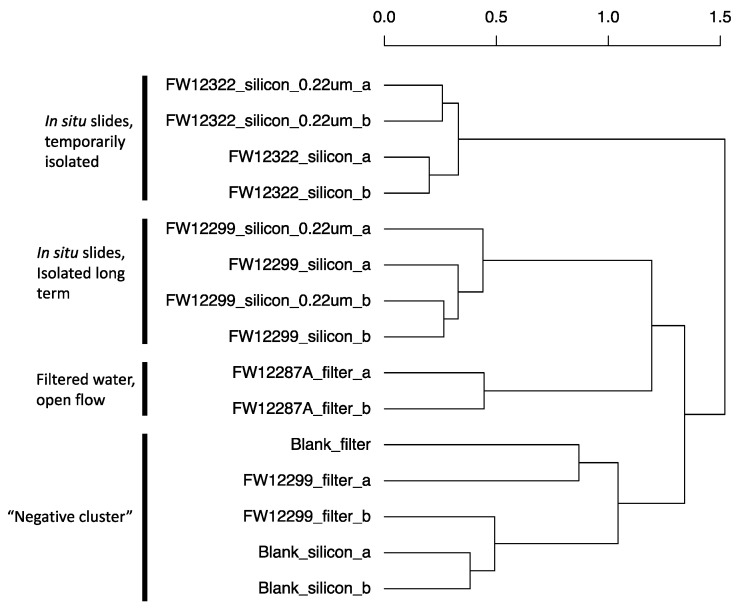
Agglomerative clustering of Kidd Creek filter and slide sample DNA results on the basis of community similarity using Ward’s method. Agglomerative coefficient = 0.73. Samples from packered boreholes isolated from the mine environment (FW12287A, FW12299) clustered together while those from a borehole freely discharging into the mine atmosphere (FW12322) clustered separately. Filtered water samples from FW12299 clustered only with negative controls, suggesting too little biomass was present to overcome background signal.

In general, Bacteroidetes were enriched in the borehole that was recently exposed to the mine environment (FW12322). Clostridia were enriched in boreholes sealed from the mine environment (FW12887A and FW12299) (Figure 4). The filter samples collected from FW12299 were most similar to the negative controls, though they did contain *Methylobacterium* spp. not seen in the control filters (Tables S2 and S3). Filters from FW12287A provided a more robust genetic signal than those from FW12299, perhaps due to the increased sediment load in the water filtered from this borehole. An agglomerative cluster analysis of the community composition in each sample shows that filter sample from FW12299 was most similar to the negative controls while FW12287A clustered with the FW12299 in situ growth slide community (Figure 5). 

The FW12287A filter samples, from the borehole that was temporarily sealed during sampling, were primarily composed of Proteobacteria (Alpha-, Beta-, and Gammaproteobacteria) and Clostridia. Actinobacteria and Bacteroidetes were also identified but did not appear in both replicates. The most abundant taxa in the filter sample from FW12287A that were not present in the negative control were primarily halophilic genera including *Marinobacter*, *Idiomarina*, *Chromohalobacter*, *Thiobacillus*, *Hyphomonas*, *Seohaeicola*, and *Fuchsiella* (Tables S2 and S4–S11). BLAST results of consensus sequences from each OTU at a species level suggest that FW12287A contains a halophilic microbial ecosystem including homoacetogenic growth on H_2_ with reduction of iron or sulfate (*Fuchsiella ferrireducens*) with heterotrophs that preferentially grow on amino acids (*Hyphomonas polymorpha*) or simple carbon compounds (*Marinobacter guineae*, *Idiomarina aestuarii*, *Seohaeicola saemankumensis*) and with some community members (*Chromohalobacter beijerinckii*) known to produce osmoprotectant extracellular molecules [46,47,48,49,50,51,52,53]. This dataset only captured cells that were entrained in water actively flowing out of FW12287A but it includes taxa not observed in service water, with the results being consistent with an ecosystem capable of supporting itself by lithoautotrophy in the absence of photosynthetic inputs.

**Figure 6:**
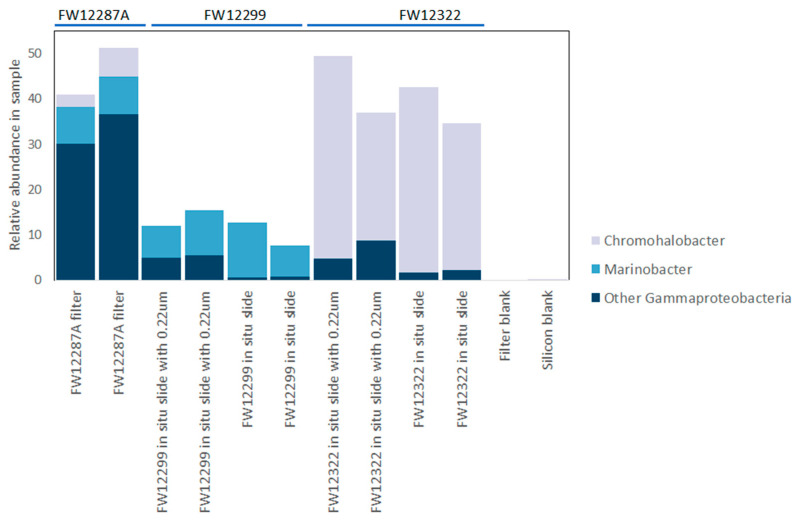
Genera within Gammaproteobacteria showing predominance of the moderately halophilic chemoorganotroph Chromohalobacter in FW12322, which has been continuously open to exchange with the mine environment as the fluids discharge.

DNA extracted from in situ growth surfaces was easily amplified, enabling full-length 16S sequencing from the smaller dataset of incubated slides. Overall, the 16S rRNA gene sequencing results were fairly diverse for both the shorter V4 sequences and for full-length 16S sequences (Figure 4 and Figure S7). Both the V4 and full-length 16S datasets showed similar taxonomic identifications but with varying relative abundances. This is not surprising given the differences in the length of amplified DNA (292 bp vs. 1466 bp) and the different biases inherent to each sequencing technology (Illumina MiSeq vs PacBio Sequel) [54]. In both cases, the vast majority of recovered taxa were known halophiles, consistent with the fracture water salinity of 2–3× that of seawater (Table 1). Both the borehole samples and negative controls were dominated by Gammaproteobacteria and Betaproteobacteria. The results from boreholes, however, included Proteobacteria taxa that differ from those of the negative controls. For example, both FW12322 and FW12299 showed evidence for *Cupriavidus*, a heterotophic soil Betaproteobacterium, and FW12299 revealed evidence for *Diaphorobacter*, a group of denitrifying Betaproteobacteria that degrade simple hydrocarbons (Table 2, Table 3 and Table S12). For different Betaproteobacteria, Suzuki et al. [55] found that their presence might reflect effective colonization and adaptation in parts of the serpentinization ecosystem of The Cedars in Northern California. FW12322 had a notable enrichment of Bacteroidetes species that was not seen in the other boreholes as well as an enrichment of the Gammaproteobacterial genus *Chromohalobacter* (Figure 6, Table S6). In contrast, FW12299 had a notable enrichment of Clostridia that was absent from FW12322 and may indicate that this isolated system, protected by the packer system, reflects a different biome potentially more reflective of the indigenous community.

The approach that was taken towards classifying the most abundant genera in each borehole focused on those taxa that were present in >1% relative abundance in the borehole and <1% abundance in the extraction and PCR negative controls, which also removed genera with <10-fold difference between the samples and controls. This eliminated 29% of reads recovered from the in situ incubation surface from FW12322 and 66.5% of reads from FW12299 (Tables S12–S18). 

The remaining abundant taxa in borehole FW12322 were strongly dominated by species most closely related to the moderately halophilic heterotrophs *Chromohalobacter salexigens* (40.5% of total sequences) and *Gracilimonas rosea* (17% of total sequences) (Table 2) [56,57]. The remaining abundant genera also best match known heterotrophs [58,59,60,61], including, notably, *Salinisphaera*, which is found in the Red Sea brine pools [58]. Given their prolonged exposure to the mine air, the microbiota of FW12322 might be more representative of the human-influenced built environment than the rock-hosted environment. Though all care was taken to maintain sterility while deploying the biosampler, the mine environment is influenced by human activity and is not expected to be pristine. The recovered biomass was also quite low, making 16S rRNA sequence detection sensitive to very low level background signals including microorganisms commonly found on human skin (e.g., *Propionibacterium acnes*). *Propionibacterium acnes* was most likely introduced to the slides at a low level via human handling after recovery of the biosamplers. Otherwise, the heterotrophs identified on the FW12322 slides were most likely resident in the borehole prior to insertion of the biosampler unit as none of the major FW12322 taxa were observed in negative controls.

At the other end of the spectrum, FW12299 had been sealed by the stainless-steel sampling manifold to isolate the borehole from the general mine environment prior to the in situ incubation. The recovered microbiota from FW12299 were more similar to the filter sample FW12287A than to the in situ incubation sample FW12322. Approximately 33.5% of genera from FW12299 were present at >1% relative abundance with <1% relative abundance in the control samples (Table 3). The species most closely related to these OTUs included sulfate-reducing bacteria (*Desulfotomaculum arcticum*), homoacetogens that can grow on H_2_ with reduction of iron or sulfate (*Fuchsiella ferrireducens*), biofilm producers that might represent low level contaminants from handling the biosamplers or from the extraction kits (*Ralstonia insidiosa*, *Propionibacterium acnes*), and heterotrophs that grow on simple organic molecules (*Caenimonas koreensis*, *Dokdonella ginsengisoli*, *Diaphorobacter nitroreducens*) and petroleum hydrocarbons (*Marinobacter gudaonensis*) [47,57,61,62,63,64,65,66]. Much like the species recovered from FW12287A filter, the species recovered from the FW12299 in situ incubation are consistent with a self-sustaining ecosystem that is supported by lithoautotrophy, consistent with both indirect and direct evidence for indigenous microbial communities in these waters [4,17].

### 3.4. Comparison to Other Subsurface Studies

Biological studies of subsurface life at Kidd Creek and in other locations have most often focused on cells collected by filtration, either by DNA analysis (e.g., [1,11,67], lipid analysis [68], or amino acid racemization [69]). These studies are instructive about the identity, quantity, and activity of planktonic life in the deep subsurface, but collecting samples from flowing water preferentially selects for motile or unbound species over biofilm-forming community members. A study of microbes cultivated within a fault zone approximately 3 km deep in a South African mine suggested that the sessile portion of a deep subsurface community can look very different from the community recovered by filtering fracture water [70]. Thus, we deployed an in situ incubation strategy to sample actively growing cells, specifically selecting for those species that grow attached to surfaces. 

The lithotrophy-supported communities from the sealed borehole 12,299 were similar to communities identified in other deep terrestrial environments. For example, *Thiobacillus denitrificans* has previously been identified in deep mines in Colorado and South Africa [11,71]. *Desulfotomaculum* spp. and other sulfate-reducing bacteria have been identified in a saline aquifer used for CO_2_ storage in Germany, in a deep gold mine in South Africa, and in a basaltic aquifer in Washington state [10,72]. Hydrocarbon-degrading halophiles including *Chromohalobacter*, *Marinobacter*, and *Ralstonia* have been identified in a hypersaline Cretaceous oil reservoir in central Africa [73]. These similarities support the finding that these components of the microbiome identified from the “corked” borehole at the Kidd Creek Observatory reflect components of indigenous microbial communities.

## 4. Conclusions

In summary, three distinct populations of organisms growing on solid surfaces within a deep mine in the Canadian Shield have been described on the basis of 16S rRNA sequences derived from filtered water and sequences extracted from in situ growth surfaces. One borehole that had been open to atmospheric exchange with the aerobic mining environment was dominated by halophilic heterotrophs. Two additional boreholes that were sealed, either for several years before sampling by a permanent stainless steel manifold (12,299), or temporarily sealed during sampling by a packer (12,287A) to reduce impact from the mining environment, contained more diverse species consistent with a community supported by lithoautotrophy and similar to species identified in other deep subsurface systems studied elsewhere. These organisms readily grew on solid surfaces with no added nutritional inputs. This work adds taxonomic information to extend the knowledge from recent work detailing the extent and activity of the indigenous microbial community in the fracture water of this deep observatory [4]. Results from this study are consistent with microbial ecosystems in deep terrestrial systems studied elsewhere in the world and provide another line of evidence that the deep terrestrial subsurface is a habitable environment that is capable of supporting life on hydrogen, methane, sulfate, and simple organic carbon molecules derived from abiotic processes. 

## Figures and Tables

**Table 1:** Historical record of physical and chemical conditions for the Kidd Creek fracture waters (from Li et. al., 2016). ND = not determined.

	FW12299 Drilled 29-May-2007					FW12287A Drilled 30-April-2007	
	27 August 2007	12 January 2010	21 October 2010	29 February 2012	14 June 2012	27 August 2007	21 October 2010
He (vol %)		2.54	2.62	2.39		2.47	
H_2_ (vol %)		4.62	3.97	3.19		10.67	
O_2_ (vol %)		0.12	<0.05	<0.05		<0.05	
N_2_ (vol %)		14.9	15.3	14.6		12.7	
CH_4_ (vol %)		71.5	70.3	71.9		72.2	
C_2_H_6_ (vol %)	ND	6.47	6.62	6.81	ND	6.56	ND
C_3_H_8_ (vol %)		0.85	0.84	0.83		0.61	
i-C_4_H_10_ (vol %)		0.07	0.07	0.07		0.04	
n-C_4_H_10_ (vol %)		0.17	0.15	0.14		0.1	
i-C_5_H_12_ (vol %)		0.05	0.06	0.05		0.03	
n-C_5_H_12_ (vol %)		0.03	0.04	0.03		0.02	
Water flow rate (mL/min)	770	172	258	170	150	79	≈5
pH	5.7	6.2	6	5.6	5.2	6.1	5.8
T (°C)	27.4	25.8	25.1	25.6	26.3	27.4	22.8
Conductivity (mS/cm)	161.1	141.3	149.6	146.8	150	109	139.9
Sulfide (μM)	<2	<2	<2	<2	<2	<2	<2
Sulfate (μM)	123	97	126	194	198	109	184

**Table 2:** Most abundant high-confidence genera in FW12322 (>1%) continuously open to atmospheric exchange with the mine environment prior to deploying the biosampler. Closest related species are halophilic heterotrophs.

Genus	FW12322 (% of Total Sequences)	Closest Related Species in RefSeq 16S Microbial Database	Identity over Full 16S Gene (%)	Negative Control, DNA Extraction Blank (%)	Negative Control, Template Free PCR (%)	Notes
*Chromohalobacter*	40.46	*Chromohalobacter salexigens*	96–99	0.01	0.23	Strictly aerobic, halophilic heterotroph [74]
*Aliifodinibius*	17.16	*Gracilimonas rosea*	88–89	0.02	0.14	Non-motile tropical marine bacterium [56,75]
*Propionibacterium*	3.20	*Propionibacterium acnes*	95–99	0.01	0.04	Common skin bacterium (most likely introduced) [57]
*Salinisphaera*	3.12	*Salinisphaera shabanensis*	94–96	0.01	0.00	halophile from Red Sea [58]
*Isosphaera*	2.96	*Isosphaera pallida*	87–89	0.15	0.01	Filamentous budding bacterium from hot springs [59]
*Cupriavidus*	2.31	*Cupriavidus basilensis*	94–98	0.00	0.00	Heterotrophic soil bacterium [60]
*Dokdonella*	1.76	*Dokdonella ginsengisoli*	97–99	0.00	0.00	Heterotrophic soil bacterium [61]

**Table 3:** Most abundant high confidence genera in FW12299 (>1%) that had previously been sealed from the mine air via a packer or “CORK”. Closest related species include halophilic sulfate-reducing bacteria, homoacetogens, and heterotrophs that specialize in degrading petroleum hydrocarbons and simple organic molecules consistent with findings from nearby fracture waters described in a recent most probable number (MPN) study [4].

Genus	FW12299 (% of Total Sequences)	Closest Related Species in RefSeq 16S Microbial Database	Identity over Full 16S Gene (%)	Negative Control, DNA Extraction Blank (%)	Negative Control, Template Free PCR (%)	Notes
*Desulfotomaculum*	6.53	*Desulfotomaculum arcticum*	91–93	0.00	0.00	Endospore-forming sulfate-reducing bacterium [62]
*Propionibacterium*	5.45	*Propionibacterium acnes*	95–99	0.01	0.04	Common skin bacterium (likely introduced) [57]
*Marinobacter*	5.22	*Marinobacter gudaonensis*	95–97	0.01	0.03	Halophile from oil-polluted saline soil [66]
*Diaphorobacter*	3.73	*Diaphorobacter nitroreducens*	97–98	0.00	0.00	Denitrifying bacterium that degrades simple hydrocarbons [65]
*Fuchsiella*	3.66	*Fuchsiella ferrireducens*	91–92	0.01	0.01	Homoacetogen capable of iron reduction [47]
*Caenimonas*	3.32	*Caenimonas koreensis*	95–96	0.00	0.03	Non-motile heterotroph from activated sludge [64]
*Ralstonia*	3.26	*Ralstonia insidiosa*	96–99	0.00	0.01	Strong biofilm producer (possible kit contaminant) [76]
*Dokdonella*	1.56	*Dokdonella ginsengisoli*	97–99	0.00	0.00	Heterotrophic soil bacterium [61]

## Supplementary Materials

The following are available online at https://www.mdpi.com/2075-1729/10/12/307/s1: Figure S1: Views of assembled and disassembled biosampler unit prior to sterilization. Figure S2: Boreholes 12299 with packer and tubing, and 12322 during and after insertion of the biosampler unit. Figure S3: 1000× phase contrast microscope images of features on glass slides from FW12299. Figure S4: Biosampler unit returned after 229 days incubation in 12299. Figure S5: SEM and EDS images of an additional FW12322 biofilm. Figure S6: SEM and EDS views of biofilm from FW12322 that is texturally similar to biofilms seen in FW12299. Figure S7: Classification of full length 16S gene OTUs for in situ incubation surfaces by phylum and class. Table S1: Geochemistry data for major cations and anions from FW12299, FW12287A, and FW12322 from the 2.4 km level of the mine. Table S2: Phylum-level results for DNA sequencing of the V4 hypervariable region of the 16S rRNA gene in both filter samples and incubated slides. Table S3: Class-level results for DNA sequencing of the V4 hypervariable region of the 16S rRNA gene in both filter samples and incubated slides. Table S4: Genera within Alphaproteobacteria found on the basis of sequencing of the V4 hypervariable region of the 16S gene in both filter samples and incubated slides. Table S5: Genera within Betaproteobacteria found on the basis of sequencing of the V4 hypervariable region of the 16S gene in both filter samples and incubated slides. Table S6: Genera within Gammaproteobacteria found on the basis of sequencing of the V4 hypervariable region of the 16S gene in both filter samples and incubated slides. Table S7: Genera within Actinobacteria found on the basis of sequencing of the V4 hypervariable region of the 16S gene in both filter samples and incubated slides. Table S8: Genera within Bacteroidetes found on the basis of sequencing of the V4 hypervariable region of the 16S gene in both filter samples and incubated slides. Table S9: Genera within Clostridia found on the basis of sequencing of the V4 hypervariable region of the 16S gene in both filter samples and incubated slides. Table S10: Phylum-level results from in situ incubations based on sequencing of the full length 16S rRNA gene. Table S11: Class-level results from in situ incubations based on sequencing of the full length 16S rRNA gene. Table S12: Most abundant genera recovered from in situ incubations based on sequencing of the full length 16S rRNA gene. Shaded boxes are sequences with >1% relative abundance in controls. Table S13: Genera within Betaproteobacteria recovered from in situ incubations based on sequencing of the full 16S gene. Table S14: Genera within Gammaproteobacteria recovered from in situ incubations based on sequencing of the full 16S gene. Table S15: Genera within Actinobacteria recovered from in situ incubations based on sequencing of the full 16S gene. Table S16: Genera within Bacteroidetes recovered from in situ incubations based on sequencing of the full 16S gene. Table S17: Genera within Clostridia recovered from in situ incubations based on sequencing of the full 16S gene. Table S18: Genera within Sphingobacteria recovered from in situ incubations based on sequencing of the full 16S gene.
