# Supplementary material for: In Situ Growth of Halophilic Bacteria in Saline Fracture Fluids from 2.4 km below Surface in the Deep Canadian Shield
Source: Life (Basel). 2020 Nov 24;10(12):307. doi: 10.3390/life10120307 (PMC7760289; doi:10.3390/life10120307)
Supplement: Supplementary file 1 [file life-10-00307-s001.pdf]

life

MDPI

Supplemental materials

In Situ Growth of Halophilic Bacteria in Saline Fractures Fluids from 2.4 km below Surface in The Deep Canadian Shield

Regina L. Wilpiszeski [1], Barbara Sherwood Lollar [2], Oliver Warr [2] and Christopher H. House [1,*]

[1] Department of Geosciences and Earth and Environmental Systems Institute, The Pennsylvania State University, University Park, PA 16802, USA; ginawilp@gmail.com
[2] Stable Isotope Laboratory, University of Toronto, Toronto, ON M5S 3B1, Canada; barbara.sherwoodlollar@utoronto.ca (B.S.L.); oliver.warr@utoronto.ca (O.W.)
* Correspondence: chrishouse@psu.edu; Tel.: 814-865-8802

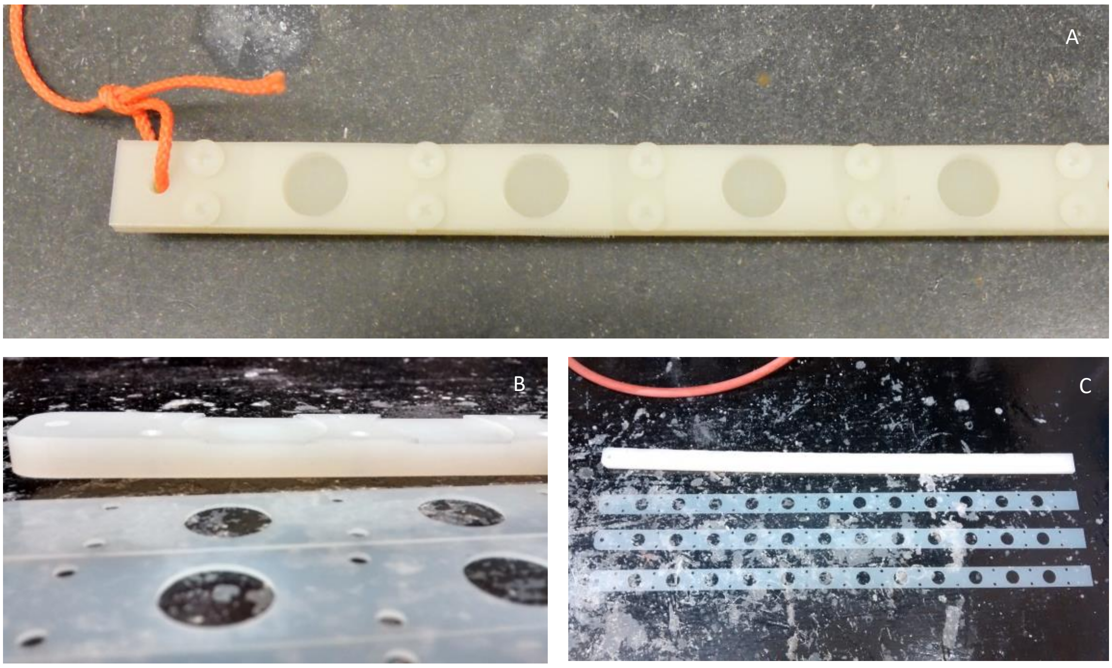

Figure S1. Views of (**A**) assembled and (**BC**) disassembled biosampler unit prior to sterilization.

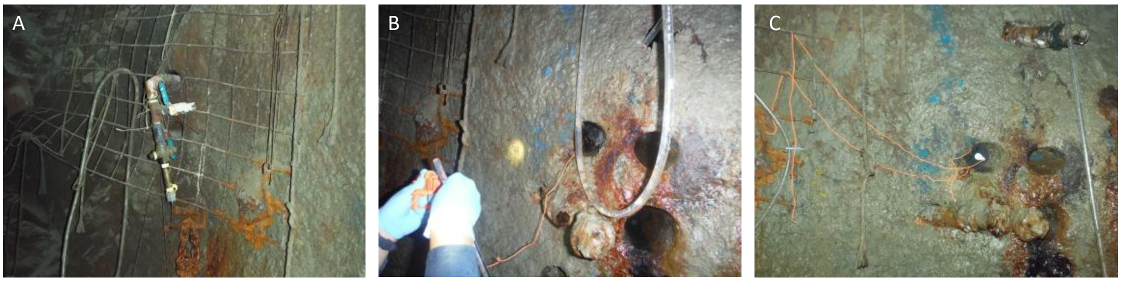

Figure S2. Boreholes (**A**) 12299 with packer and tubing, and 12322 (**B**) during and (**C**) after insertion of the biosampler unit.

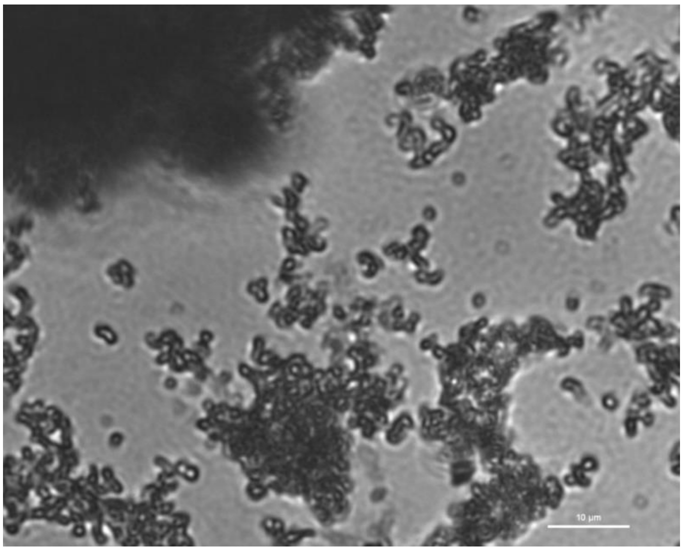

Figure S3. 1000× phase contrast microscope images of presumed-precipitate features on glass slides from FW12299. Similar structures were seen on glass slides from FW12322 and controls incubated with filtered water from FW12299.

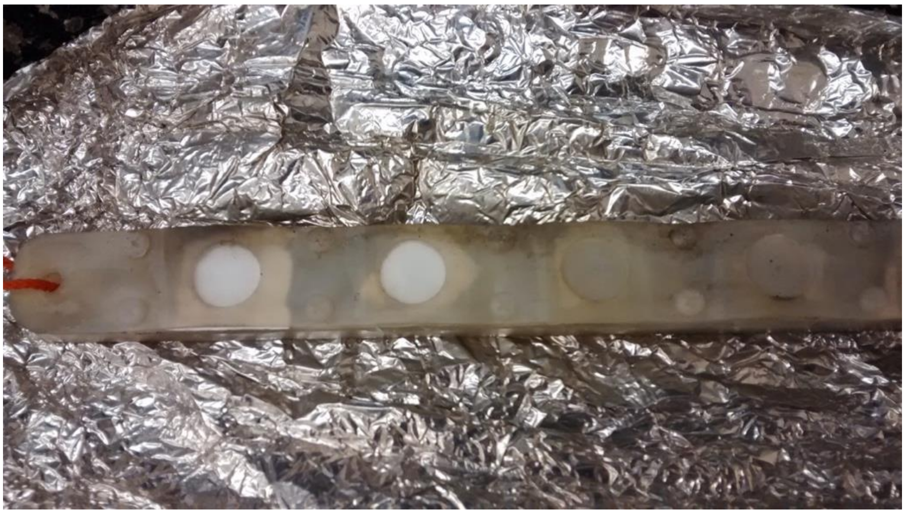

Figure S4. Biosampler unit returned after 229 days incubation in 12299. Pictured are two 1″ round slides with 0.2 um size exclusion filter (**left**) and two without (**right**).

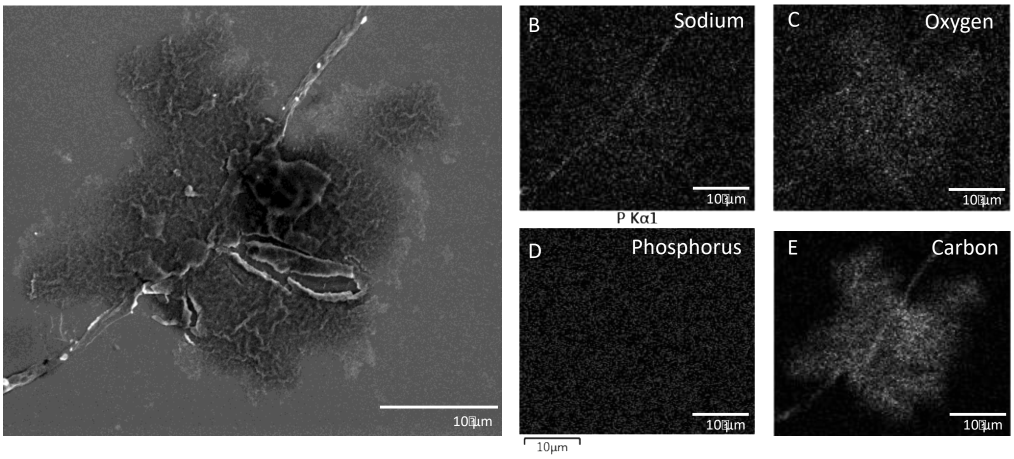

Figure S5. (**A**) SEM and (**B–E**) EDS images of an additional FW12322 biofilm.

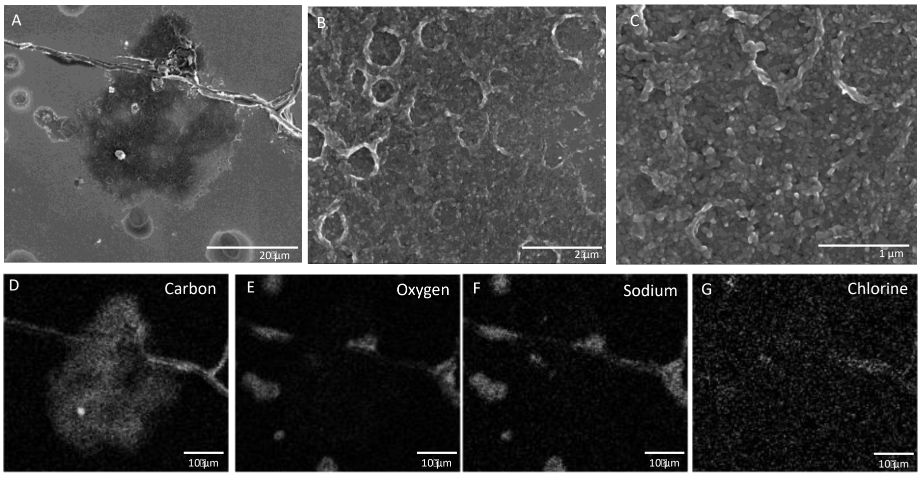

Figure S6. (A-C) SEM and (D-G) EDS views of biofilm from FW12322 that is texturally similar to biofilms seen in FW12299. FW12322 was open to exchange with the mine environment prior to biofilm insertion.

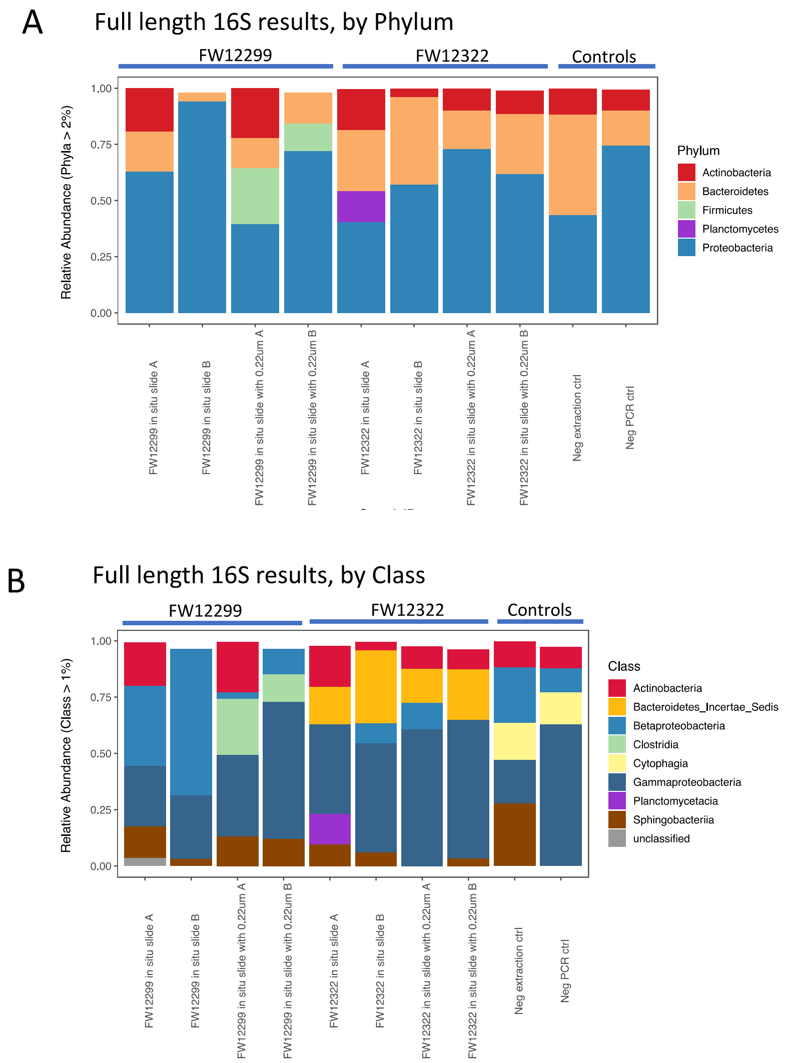

Figure S7. Classification of full length 16S gene OTUs for in situ incubation surfaces by (A) phylum and (B) class. Results are more variable than those obtained by MiSeq sequencing but still show a strong enrichment of Bacteroidetes in boreholes recently exposed to the mine (FW12322) and the presence of Clostridia in the packered ("corked") borehole (FW12299).

Table S1. Geochemistry data for major cations and anions from FW12299, FW12287A, and FW12322 from the 2.4 km level of the mine.

Geochemical compositions (mol/L)	FW12299	FW12287A	FW12322
Na^+ (mol/L)	6.7E-01	5.6E-01	7.2E-01
Total Fe (mol/L)	2.3E-04	1.1E-04	1.5E-04
K^+ (mol/L)	4.2E-03	2.8E-03	4.9E-03
Mg^{2+} (mol/L)	9.9E-02	1.1E-01	7.7E-02
Ca^{2+} (mol/L)	1.3E+00	9.8E-01	1.6E+00
Cl^- (mol/L)	3.4E+00	2.7E+00	4.0E+00
Br^- (mol/L)	2.7E-02	1.9E-02	2.8E-02
NO_3^- (mol/L)	<1.8E-04	<1.8E-04	<3.2E-04
NO_2^- (mol/L)	<2.2E-04	<2.2E-04	<4.3E-04
HPO_4^{2-} (mol/L)	<3.3E-04	<3.3E-04	<4.2E-04
SO_4^{2-} (mol/L)	3.3E-04	3.9E-04	<6.2E-04
Tot salinity (mol/L)	5.4E+00	4.4E+00	6.4E+00
TDS (g/L)	190	153	227
Charge Balance err	0.8%	-0.1%	0.0%

Table S2. Phylum level results for DNA sequencing of the V4 hypervariable region of the 16S rRNA gene in both filter samples and incubated slides.

Phylum	FW12299 slide (%)	FW12322 slide (%)	Slide neg. ctrl (%)	FW12299 filter (%)	FW12287 A filter (%)	Filter neg. ctrl (%)
Proteobacteria	40.62	63.94	72.16	99.71	73.11	85.87
Bacteroidetes	1.09	27.14	0.57	-	3.86	2.63
Firmicutes	57.08	8.33	21.94	0.07	18.80	9.19
Actinobacteria	1.15	0.51	5.29	0.22	1.80	-
Verrucomicrobia	-	0.05	-	-	-	-
Unclassified Bacteria	0.05	0.02	0.01	-	0.09	-
Euryarchaeota	-	0.01	-	-	0.33	-
Elusimicrobia	-	0.01	-	-	-	-
Acidobacteria	-	-	0.01	-	-	-
Chlamydiae	-	-	-	-	0.05	-
Chloroflexi	-	-	-	-	-	-
Deinococcus-Thermus	-	-	-	-	0.66	2.31
Fusobacteria	-	-	0.03	-	-	-
Gemmatimonadetes	-	-	-	-	0.02	-
Nitrospirae	-	-	-	-	-	-
Planctomycetes	-	-	-	-	0.81	-
Saccharibacteria	-	-	-	-	0.48	-

Table S3. Class level results for DNA sequencing of the V4 hypervariable region of the 16S rRNA gene in both filter samples and incubated slides.

Class	FW12299 slide (%)	FW12322 slide (%)	Slide ctrl (%)	FW12299 filter (%)	FW12287 A filter (%)	Filter ctrl (%)
Clostridia	37.39	0.04	0.06	0.02	18.21	-
Betaproteobacteria	26.02	22.10	71.55	63.67	16.85	85.84
Bacilli	19.68	8.29	21.87	0.05	0.57	9.19
Gammaproteobacteria	12.27	40.14	0.05	0.02	45.69	-
Alphaproteobacteria	2.28	1.67	0.55	36.03	9.80	-
Actinobacteria	1.15	0.51	5.29	0.22	1.71	-
Flavobacteriia	0.60	-	-	-	1.02	2.63
Sphingobacteriia	0.48	-	0.55	-	0.27	-
Unclassified Bacteria	0.05	0.02	0.01	-	0.09	-
Deltaproteobacteria	0.03	-	-	-	0.75	0.02
Unclassified Proteobacteria	0.02	0.02	-	-	0.03	-
Unclassified Firmicutes	0.01	-	-	-	0.02	-
Bacteroidia	0.01	0.03	0.01	-	0.11	-
Bacteroidetes	-	27.11	-	-	1.82	-
Ktedonobacteria	-	-	-	-	-	-
Nitrospira	-	-	-	-	-	-
Halobacteria	-	0.01	-	-	0.33	-
Holophagae	-	-	0.01	-	-	-
Acidimicrobiia	-	-	-	-	0.08	-
Thermoleophilia	-	-	-	-	0.02	-
Cytophagia	-	-	-	-	0.65	-
Chlamydiae	-	-	-	-	0.05	-
Deinococci	-	-	-	-	0.66	2.31
Elusimicrobia	-	0.01	-	-	-	-
Fusobacteriia	-	-	0.03	-	-	-
Gemmatimonadetes	-	-	-	-	0.02	-
Planctomycetacia	-	-	-	-	0.81	-
Epsilonproteobacteria	-	0.01	0.01	-	-	-
Saccharibacteria	-	-	-	-	0.48	-
Unclassified Verrucomicrobia	-	0.05	-	-	-	-

Table S4. Genera within Alphaproteobacteria based on sequencing of the V4 hypervariable region of the 16S gene in both filter samples and incubated slides.

Genera in Alphaproteobacteria	FW12299 slide (%)	FW12322 Slide (%)	Slide ctrl (%)	FW12299 filter (%)	FW12287 A filter (%)	Filter ctrl (%)
(Number of sequences)	*(713)*	*(289)*	*(95)*	*(2110)*	*(653)*	*(0)*
Unclassified Hyphomicrobiaceae	35.48	-	-	-	-	-
Aquamicrobium	18.37	6.38	3.16	2.94	0.15	-
Pelagibacterium	17.67	-	-	-	-	-
Unclassified Sphingomonadales	10.80	6.57	-	0.09	12.10	-
Unclassified Rhodobacteraceae	10.52	-	-	-	6.74	-
Blastomonas	5.05	-	-	-	-	-
Seohaeicola	0.70	-	-	-	16.39	-
Unclassified Sphingomonadaceae	0.56	62.63	-	2.61	-	-
Unclassified Rhizobiales	0.42	19.38	4.21	0.19	1.99	-
Methylobacterium	0.14	1.73	91.58	94.17	-	-
Rhizobium	0.14	-	-	-	-	-
Unclassified Alphaproteobacteria	0.14	-	-	-	0.46	-
Brevundimonas	-	2.08	-	-	-	-
Phenylobacterium	-	-	-	-	1.23	-
Hyphomonas	-	0.69	-	-	47.93	-
Salinarimonas	-	-	-	-	2.60	-
Brucella	-	0.35	-	-	-	-
Parvibaculum	-	-	-	-	8.42	-
Unclassified Xanthobacteraceae	-	-	1.05	-	-	-
Candidatus_Odyssella	-	-	-	-	1.99	-

Table S5. Genera within Betaproteobacteria based on sequencing of the V4 hypervariable region of the 16S gene in both filter samples and incubated slides.

Genera in Betaproteobacteria	FW12299 slide (%)	FW12322 slide (%)	Slide ctrl (%)	FW12299 filter (%)	FW12287A filter (%)	Filter ctrl (%)
(Number of sequences)	*(8135)*	*(3825)*	*(12388)*	*(3729)*	*(1123)*	*(3784)*
Unclassified Alcaligenaceae	80.06	93.59	99.07	99.97	56.19	29.89
Thiobacillus	7.49	-	-	0.03	22.26	-
Unclassified Burkholderiaceae	5.38	0.03	0.01	-	7.12	3.49
Unclassified Comamonadaceae	2.57	0.52	0.02	-	5.88	0.50
Ralstonia	1.84	0.08	0.48	-	-	66.12
Burkholderia-Paraburkholderia	1.83	3.63	-	-	-	-
Alcaligenes	0.55	1.20	0.03	-	1.34	-
Aquabacterium	0.20	-	-	-	-	-
Unclassified Oxalobacteraceae	0.06	-	-	-	7.03	-
Massilia	0.01	0.44	0.36	-	-	-
Cupriavidus	-	0.34	-	-	-	-
Roseateles	-	-	0.03	-	-	-
Janthinobacterium	-	0.13	-	-	-	-
Uncultured Rhodocyclaceae	-	0.03	-	-	-	-
Unclassified Betaproteobacteria	-	-	-	-	0.18	-

Table S6. Genera within Gammaproteobacteria based on sequencing of the V4 hypervariable region of the 16S gene in both filter samples and incubated slides.

Genera in Gammaproteobacteria	FW12299 slide (%)	FW12322 slide (%)	Slide ctrl (%)	FW12299 filter (%)	FW12287A filter (%)	Filter ctrl (%)
(Number of sequences)	*(3836)*	*(6948)*	*(8)*	*(1)*	*(3045)*	*(0)*
Marinobacter	77.97	-	12.50	-	18.26	-
Unclassified Gammaproteobacteria	6.00	0.09	-	-	2.46	-
Unclassified Pseudomonadaceae	4.54	0.35	75.00	100.00	0.76	-
Alishewanella	3.62	-	-	-	-	-
Acinetobacter	2.35	0.19	-	-	1.61	-
Idiomarina	1.88	-	-	-	15.60	-
Unclassified Halomonadaceae	1.38	1.81	-	-	10.38	-
Alkanindiges	1.07	-	-	-	-	-
Stenotrophomonas	0.81	0.06	-	-	-	-
Salinicola	0.31	-	-	-	-	-
Unclassified Alishewanella	0.05	-	-	-	0.16	-
Unclassified Oceanospirillales	0.03	-	-	-	0.03	-
1013-28-CG33	-	-	-	-	0.23	-
34P16	-	-	-	-	0.23	-
Unclassified Idiomarinaceae	-	-	-	-	0.07	-
Marinimicrobium	-	-	-	-	0.10	-
Unclassified Cellvibrionales	-	-	-	-	2.66	-
Halothiobacillus	-	-	-	-	2.66	-
Thiovirga	-	-	-	-	0.85	-
Unclassified Halothiobacillaceae	-	-	-	-	0.03	-
Unclassified Enterobacteriaceae	-	0.35	-	-	0.03	-
Legionella	-	-	-	-	0.19	-
Alcanivorax	-	-	-	-	8.90	-
Kangiella	-	-	-	-	2.07	-
Chromohalobacter	-	89.91	12.50	-	9.52	-
Oceanobacter	-	-	-	-	0.03	-
Pseudohongiella	-	-	-	-	0.10	-
Enhydrobacter	-	-	-	-	0.10	-
Perlucidibaca	-	-	-	-	0.33	-
unclassified	-	-	-	-	0.03	-
Salinisphaera	-	7.25	-	-	10.84	-
EV818SWSAP88_ge	-	-	-	-	0.23	-
Methylophaga	-	-	-	-	2.07	-
Thioalkalimicrobium	-	-	-	-	3.61	-
Arenimonas	-	-	-	-	0.10	-
Luteimonas	-	-	-	-	0.82	-
Silanimonas	-	-	-	-	2.99	-
unclassified	-	-	-	-	1.94	-

Table S7. Genera within Actinobacteria based on sequencing of the V4 hypervariable region of the 16S gene in both filter samples and incubated slides.

**Genera in Actinobacteria**	**FW12299 slide (%)**	**FW12322 slide (%)**	**Slide neg. ctrl. (%)**	**FW12299 filter (%)**	**FW12287A filter (%)**	**Filter neg. ctrl. (%)**
(Number of sequences)	*(360)*	*(89)*	*(916)*	*(13)*	*(120)*	*(0)*
Rhodococcus	50.83	-	-	-	-	
Micrococcus	26.11	-	-	-	-	-
Unclassified Microbacteriaceae	10.83	95.51	98.91	100.00	45.00	-
Unclassified Micrococcales	9.17	3.37	0.55	-	-	-
Unclassified Mycobacterium	1.94	-	-	-	3.33	-
Unclassified Corynebacteriales	0.56	-	-	-	-	-
Unclassified Nocardiaceae	0.28	-	-	-	-	-
Unclassified Actinobacteria	0.28	-	0.11	-	-	-
Iamia	-	-	-	-	1.67	-
OM1_clade_ge	-	-	-	-	2.50	-
Actinomyces	-	-	-	-	2.50	-
Gardnerella	-	-	-	-	2.50	-
Corynebacterium	-	-	-	-	7.50	-
Uncultured Frankiales	-	1.12	-	-	-	-
Unclassified Micrococcales	-	-	-	-	2.50	-
Nocardioides	-	-	-	-	31.66	-
Unclassified Pseudonocardiaceae	-	-	0.44	-	-	-
Gaiella	-	-	-	-	0.83	-

Table S8. Genera within Bacteroidetes based on sequencing of the V4 hypervariable region of the 16S gene in both filter samples and incubated slides.

Genera in Bacteroidetes	FW12299 slide (%)	FW12322 slide (%)	Slide neg. ctrl (%)	FW12299 filter (%)	FW12287A filter (%)	Filter neg. ctrl (%)
(Number of sequences)	*(342)*	*(4697)*	*(98)*	*(125)*	*(257)*	*(116)*
Unclassified Sphingobacteriales	43.70	-	97.96	-	-	-
Flavobacterium	31.09	-	-	-	-	-
Unclassified Flavobacteriaceae	24.05	-	-	-	0.39	-
Alistipes	0.59	0.02	-	-	-	-
CK06-06-Mud-MAS4B-21	0.29	-	-	-	29.18	-
Bacteroidales_S24-7 group	0.29	0.09	2.04	-	-	-
Aliifodinibius	-	0.15	-	-	-	-
Gracilimonas	-	-	-	-	1.56	-
Unknown_Family	-	0.02	-	-	-	-
Unclassified Bacteroidetes	-	99.72	-	-	16.73	-
Meniscus	-	-	-	-	2.33	-
Fontibacter	-	-	-	-	8.17	-
Unclassified Cyclobacteriaceae	-	-	-	-	8.56	-
Arenibacter	-	-	-	-	3.11	-
Bergeyella	-	-	-	-	21.79	-
Capnocytophaga	-	-	-	-	1.17	-
Cloacibacterium	-	-	-	-	-	100.00
Pedobacter	-	-	-	-	7.00	-

Table S9. Genera within Clostridia based on sequencing of the V4 hypervariable region of the 16S gene in both filter samples and incubated slides.

Genera in Clostridia	FW12299 slide (%)	FW12322 slide (%)	Slide ctrl. (%)	FW12299 filter (%)	FW12287A filter (%)	Filter ctrl. (%)
(Number of sequences)	*(11688)*	*(7)*	*(11)*	*(1)*	*(1214)*	*(0)*
Fuchsiella	99.80	14.29	9.09	100.00	94.98	-
Unclassified Clostridia	0.08	-	-	-	0.16	-
Uncultured Lachnospiraceae	0.06	42.86	72.73	-	3.05	-
Proteiniclasticum	0.03	-	-	-	-	-
Lachnospiraceae_NK4A136_group	0.02	-	18.18	-	-	-
Unclassified Clostridiales	0.01	-	-	-	-	-
Lachnoclostridium	0.01	-	-	-	-	-
Lachnospiraceae_UCG-001	-	14.29	-	-	-	-
Uncultured Peptococcaceae	-	14.29	-	-	-	-
Ruminiclostridium_9	-	14.29	-	-	-	-
SRB2	-	-	-	-	0.91	-

Table S10. Phylum level results from *in situ* incubations based on sequencing of the full length 16S rRNA gene.

Phylum	FW12299 (%)	FW12322 (%)	Neg. ctrl. extraction (%)	Neg. ctrl. PCR (%)
(Number of sequences)	*(35038)*	*(35649)*	*(9744)*	*(7811)*
Proteobacteria	65.52	59.59	43.66	74.38
Bacteroidetes	12.65	27.00	44.61	15.58
Actinobacteria	11.43	10.15	11.51	9.35
Firmicutes	10.34	0.12	0.04	0.01
Planctomycetes	0.05	2.81	0.15	0.68
Unclassified Bacteria	0.01	-	0.02	-
Armatimonadetes	-	0.11	-	-
Chloroflexi	-	0.04	-	-
Deinococcus-Thermus	-	0.17	-	-

Table S11. Class level results from *in situ* incubations based on sequencing of the full length 16S rRNA gene.

Class	FW12299 (%)	FW12322(%)	Neg. ctrl. extraction (%)	Neg. ctrl. PCR (%)
(Number of sequences)	*(35038)*	*(35649)*	*(9744)*	*(7811)*
Gammaproteobacteria	39.69	53.49	19.15	62.80
Betaproteobacteria	25.36	5.80	24.51	10.70
Actinobacteria	11.43	9.76	11.51	9.35
Sphingobacteriia	11.07	4.90	27.91	0.92
Clostridia	10.20	-	-	-
Unclassified Bacteroidetes	0.92	0.01	-	-
Cytophagia	0.55	0.06	16.63	14.48
Alphaproteobacteria	0.47	0.31	-	0.88
Bacilli	0.14	0.12	-	-
Flavobacteriia	0.08	0.52	-	-
Planctomycetacia	0.05	2.81	-	0.68
Bacteroidetes Incertae Sedis	0.02	21.51	-	-
Unclassified Bacteria	0.01	0.00	-	-
Acidimicrobiia	-	0.39	-	-
unclassified	-	0.00	-	-
Fimbriimonadia	-	0.11	-	-
Thermomicrobia	-	0.04	-	-
Deinococci	-	0.17	-	-
Unclassified Proteobacteria	-	-	-	-

Table S12. Most abundant genera recovered from *in situ* incubations based on sequencing of the full length 16S rRNA gene. Shaded boxes are sequences with > 1% rel. abund. in controls.

Genus	FW12299 (%)	FW12322 (%)	Neg. ctrl. extraction (%)	Neg. ctrl. PCR (%)
Chromohalobacter	0.03	40.46	0.01	0.23
Unclassified Aliifodinibius	0.02	17.16	0.02	0.14
Propionibacterium	5.45	3.20	0.01	0.04
Salinisphaera	0.01	3.12	0.01	0.00
Isosphaera	0.00	2.96	0.15	0.01
Cupriavidus	0.72	2.31	0.00	0.00
Dokdonella	1.56	1.76	0.00	0.00
Unclassified Caenimonas	3.32	0.03	0.00	0.03
Marinobacter	5.22	0.00	0.01	0.03
Diaphorobacter	3.73	0.00	0.00	0.00
Desulfotomaculum	6.53	0.00	0.00	0.00
Fuchsiella	3.66	0.00	0.01	0.01
Ralstonia	3.26	0.00	0.00	0.01
Pseudomonas	31.83	9.38	13.32	60.16
Unclassified Oxalobacteraceae	8.67	2.39	1.27	10.32
Mucilaginibacter	8.41	3.94	26.23	0.87
Massilia	5.30	0.01	23.18	0.03
Pseudonocardia	4.05	2.48	1.84	2.87
Taibaiella	2.61	0.00	1.53	0.05
Actinoplanes	0.54	2.66	0.03	1.51
Nocardioides	0.00	2.23	4.44	2.47

Table S13. Genera within Betaproteobacteria recovered from *in situ* incubations based on sequencing of the full 16S gene.

Genera in Betaproteobacteria	FW12299 (%)	FW12322 (%)	Neg. ctrl. reaction (%)	Neg. ctrl. PCR (%)
(Number of sequences)	*(8887)*	*(2068)*	*(2388)*	*(836)*
Unclassified Oxalobacteraceae	34.36	68.38	5.19	97.37
Massilia	20.88	0.10	94.60	0.24
Diaphorobacter	14.71	0.05	-	-
Unclassified Comamonadaceae	13.11	0.39	-	0.24
Ralstonia	12.85	-	-	0.12
Cupriavidus	2.85	29.93	-	-
Unclassified Burkholderiales	0.83	1.02	-	1.56
Caenimonas	0.12	-	-	-
Rugamonas	0.12	-	0.04	0.12
Thauera	0.09	-	-	-
Paraherbaspirillum	0.02	0.10	-	0.24
Uncultured Burkholderiales	0.02	-	-	0.12
Unclassified Betaproteobacteria	0.02	0.05	-	-
Pseudoduganella	-	-	0.17	-

Table S14. Genera within Gammaproteobacteria recovered from *in situ* incubations based on sequencing of the full 16S gene.

Genera in Gammaproteobacteria	FW12299 (%)	FW12322 (%)	Neg. ctrl. extraction (%)	Neg. ctrl. PCR (%)
(Number of sequences)	*(13905)*	*(19067)*	*(1866)*	*(4905)*
Pseudomonas	80.19	22.89	69.56	95.80
Marinobacter	13.15	0.01	0.05	0.04
Dokdonella	3.94	2.48	-	-
Perlucidibaca	1.66	-	-	-
Uncl. Pseudomonadaceae	0.86	0.19	0.27	0.77
Chromohalobacter	0.09	68.82	0.05	0.37
Uncl. Gammaproteobacteria	0.04	-	-	0.04
Acinetobacter	0.04	-	0.32	-
Salinisphaera	0.01	4.98	0.05	-
Thiovirga	0.01	-	-	-
Azorhizophilus	0.01	-	0.05	-
Uncl. Xanthomonadaceae	0.01	0.09	-	-
Aidingimonas	-	-	0.05	-
Uncl. Halomonadaceae	-	0.40	-	0.02
Luteimonas	-	0.25	0.05	-
Steroidobacter	-	-	29.53	2.96

Table S15. Genera within Actinobacteria recovered from *in situ* incubations based on sequencing of the full 16S gene.

Genera in Actinobacteria	FW12299 (%)	FW12322 (%)	Neg. ctrl. extraction (%)	Neg. ctrl. PCR (%)
(Number of sequences)	*(4006)*	*(3619)*	*(1122)*	*(730)*
Propionibacterium	47.70	23.71	0.09	0.41
Pseudonocardia	35.42	18.40	15.95	30.68
Actinoplanes	4.72	20.14	0.27	16.16
Uncl. Micromonosporaceae	3.20	9.26	20.86	13.84
Mycobacterium	2.50	-	-	-
Virgisporangium	2.50	-	-	-
Micromonospora	1.92	5.19	22.19	8.90
Cryptosporangium	1.17	0.06	0.18	0.41
Micromonosporacea	0.30	1.05	1.60	2.88
Uncl. Pseudonocardiaceae	0.25	0.33	-	0.27
Uncl. Actinobacteria	0.15	0.06	-	-
Uncl. Corynebacteriales	0.07	-	-	-
Unclassified	0.07	-	-	-
Xiangella	0.02	-	0.09	-
Uncl. Acidimicrobiales	-	0.06	-	-
Uncultured Acidimicrobiales	-	3.79	-	-
Nocardioides	-	17.93	38.77	26.44
Uncl. Actinobacteria	-	0.03	-	-

Table S16. Genera within Bacteroidetes recovered from in situ incubations based on sequencing of the full 16S gene. *Top Blast hits have 88% identity to *Gracilimonas rosea.* **Top Blast hits have 90-95% identity to *Aliifodinibius roseus.*

Genera in Bacteroidetes	FW12299 (%)	FW12322 (%)	Neg. ctrl. Extraction (%)	Neg. ctrl. PCR (%)
(Number of reads)	*(7)*	*(7669)*	*(3)*	*(12)*
Unclassified Bacteroidetes Incertae Sedis	100*	96.24**	66.67*	91.67*
Aliifodinibius	-	3.76	33.33	8.33
Fodinibius	-	0.01	-	-

Table S17. Genera within Clostridia recovered from *in situ* incubations based on sequencing of the full 16S gene.

Genera in Clostridia	FW12299 (%)	FW12322 (%)	Neg. ctrl. extraction (%)	Neg. ctrl. PCR (%)
(Number of sequences)	*(3574)*	*(0)*	*(1)*	*(1)*
Sporotomaculum	64.02	-	-	-
Fuchsiella	35.93	-	100	100
Uncl. Halobacteroidaceae	0.06	-	-	-

Table S18. Genera within Sphingobacteria recovered from *in situ* incubations based on sequencing of the full 16S gene.

Genera in Sphingobacteriia	FW12299 (%)	FW12322 (%)	Neg. ctrl. extraction (%)	Neg. ctrl. PCR (%)
(Number of sequences)	*(3880)*	*(1195)*	*(2720)*	*(72)*
Mucilaginibacter	75.93	88.37	93.97	94.44
Taibaiella	23.53	-	5.48	5.56
Terrimonas	0.23	11.05	-	-
Unclassified Sphingobacteriaceae	0.16	0.42	0.37	-
Uncl. Chitinophagaceae	0.08	0.17	-	-
Parasediminibacterium	0.03	-	-	-
S15-21	0.03	-	-	-
Filimonas	-	-	0.18	-

Publisher's Note: MDPI stays neutral with regard to jurisdictional claims in published maps and institutional affiliations.

© 2020 by the authors. Submitted for possible open access publication under the terms and conditions of the Creative Commons Attribution (CC BY) license (http://creativecommons.org/licenses/by/4.0/).
